# Impact of initial hematocrit levels during direct procurement and perfusion on the likelihood of primary graft dysfunction in donation after circulatory death heart transplantation

**DOI:** 10.1016/j.jhlto.2025.100442

**Published:** 2025-11-15

**Authors:** Emmanuel Odekunle, Adham Makarem, George Olverson, Kamila Drezek, Eriberto Michel, David D’Alessandro, Ioannis Mastoris, Shannon N. Tessier, Asishana Osho, S. Alireza. Rabi

**Affiliations:** a Division of Cardiac Surgery, Massachusetts General Hospital, Harvard Medical School, Boston, Massachusetts; b Rutgers New Jersey Medical School, Newark, New Jersey; c University of Rochester School of Medicine and Dentistry, Rochester, New York; d Department of Cardiology, Massachusetts General Hospital, Harvard Medical School, Boston, Massachusetts; e Center for Engineering in Medicine and Surgery, Massachusetts General Hospital, Harvard Medical School, Shriners Children's Boston, Boston, Massachusetts

**Keywords:** donation after circulatory death, direct procurement and perfusion, hematocrit, primary graft dysfunction, extracorporeal membrane oxygenation, ex vivo perfusion, perfusate

## Abstract

**Background:**

Optimizing perfusion parameters when using ex vivo heart perfusion systems is critical for maximizing organ quality and extending perfusion duration. Hematocrit (HCT) of the perfusate may influence oxygen delivery and perfusion dynamics, yet its clinical significance remains uncertain.

**Methods:**

In this retrospective single-center study, we utilize regression models to explore the relationship between hematocrit (HCT) of the device perfusate during normothermic machine perfusion (using the Transmedics Organ System [OCS]) and recipient outcomes following transplantation. Cohorts with HCT <15% packed cell volume [PCV] were compared with those with HCT ≥15% PCV.

**Results:**

Among 74 recipients, moderate and severe primary graft dysfunction (defined as needing extracorporeal machine oxygenation post-transplant) did not differ significantly between cohorts. Mean inotrope scores 48 hours post admission were significantly higher in recipients with higher perfusate HCT. The ICU length of stay in the higher HCT group was numerically high but did not achieve statistical significance.

**Conclusion:**

Higher perfusate HCT was not associated with reduced PGD and may be linked to increased postoperative support requirements. These findings raise important questions about optimal HCT levels for ex vivo perfusion and warrant further prospective investigation.

## Background

Donation after circulatory death (DCD) heart transplantation presents challenges due to warm ischemia time (WIT), which some studies suggest is associated with increased risk of primary graft dysfunction (PGD).^1^, ^2^ To mitigate these risks, many centers have adopted the direct procurement and perfusion method, wherein the heart is perfused ex vivo using normothermic, oxygenated donor blood. This perfusate is crucial for maintaining the heart’s vitality during the procurement process, as it is purported to provide adequate oxygen, nutrition, and metabolic substrates.^3^ This study aims to investigate whether increased hematocrit (HCT) levels in the perfusate might offer protective effects by reducing the likelihood of PGD, among other outcomes, in DCD heart transplant recipients.

## Methods

This single-center retrospective study examined DCD heart transplants performed at Massachusetts General Hospital between December 2019 and January 2023. Hearts were procured using the Transmedics OCS ex vivo normothermic perfusate system, and perfusate HCT was measured at time of sampling via i-STAT CG8+ analysis within 10 minutes of perfusion initiation. These printouts do not provide measurements of free heme. Hearts were stratified into 2 groups: HCT ≥15% packed cell volume (PCV) and HCT <15% PCV, with 15% as the lower limit of the i-STAT analysis.

Outcomes analyzed included PGD incidence and extracorporeal machine oxygenation (ECMO) utilization. Secondary outcomes were intensive care unit (ICU) and hospital length of stay, pre- and post-transplant arterial and venous lactate levels, and inotropic support scores at ICU admission and 48 hours post admission. PGD and inotropes, as calculated below, were defined per International Society for Heart and Lung Transplant consensus guidelines.^4^, ^5^ Inotropes were weaned primarily based on achieving a cardiac index >2.2 liter/min/m^2^. Additional criteria included a serum lactate <2.0 mmol/liter and clinical signs of adequate tissue perfusion. Vasopressors were weaned based on achieving a mean arterial pressure ≥65 mm Hg. Perioperative care protocols were standardized to minimize variability. A multidisciplinary heart failure team rounded on patients daily and applied these standardized protocols, which helped ensure consistency. Although physician discretion remained a factor, adherence to the established criteria supported uniformity between groups.

Inotrope score = dopamine (×1) + dobutamine (×1) + amrinone (×1) + milrinone (×15) + epinephrine (×100) + norepinephrine (×100) 67 with each drug dosed in μg/kg/min

Continuous variables are presented as medians with interquartile ranges or means with standard deviations, while categorical variables are reported as frequencies with percentages. Continuous variables were compared using the Wilcoxon Rank Sum test or the independent samples *t*-test, depending on normality assessed via histograms. Categorical variables were analyzed using the chi-square test or Fisher’s exact test. HCT was treated as a binary variable. Associations with study outcomes were assessed using simple linear regression for continuous outcomes and univariate logistic regression for binary outcomes. To assess for potential timeline, we examined the perfusate HCT levels distribution over time and discovered no systematic trend that would be indicative of earlier transplants receiving higher or lower HCT perfusates thus reducing the likelihood of a confounding learning curve or evolution of protocol.

The analysis included less than 5% missing data, addressed via complete case analysis. Statistical significance was set at a *p*-value of <0.05. Analyses were performed using SAS 9.4 (SAS Institute, Cary, NC).

## Results

Seventy-four DCD heart transplant recipients (HCT < 15% PCV: N = 25; HCT ≥ 15% PCV: N = 49) were included with findings reported in Tables 1 and 2. Baseline donor and recipient demographics were similar between groups (Table 1). Procedure and hemodynamic characteristics 24 hours post ICU admission were not statistically different between cohorts (Table 2).

**Table 1:** Baseline Donor and Recipient Demographics in PGD Following DCD Heart Transplantation

Variables	HCT < 15 (*n* = 25)	HCT ≥ 15 (*n* = 49)	*p*-value
*Recipient characteristics*			
Age, year, median [Interquartile Range (IQR)]	55 [44, 63]	57 [47, 62]	0.6985
Male, sex, *n* (%)	18 (72)	39 (79.6)	0.5615
Height, m	1.7335 (0.092)	1.7496 (0.077)	0.4315
Weight, kg	82.8 (15.3)	86.4 (14.4)	0.3205
Body Mass Index (BMI), kg/m^2^	27.5 (4.5)	28.2 (4.2)	0.5013
Blood group			0.2653
O	14 (56)	29 (59.2)	
A	11 (44)	16 (32.7)	
B	0 (0)	4 (8.1)	
AB	0 (0)	0 (0)	
Race			0.4717
White	20 (80)	39 (79.6)	
Black	3 (12)	5 (10.2)	
Other	2 (8)	5 (10.2)	
Cardiac disease etiology			0.1571
Nonischemic dilated cardiomyopathy	12 (48)	26 (53)	
Ischemic dilated cardiomyopathy	9 (36)	16 (32.7)	
Other	4 (16)	7 (14.3)	
Durable left ventricular assist device	14 (56)	24 (49)	0.8908
Chronic kidney disease	9 (36)	21 (42.9)	0.6239
Diabetes mellitus	10 (40)	18 (36.7)	0.7166
Hyperlipidemia	14 (56)	23 (46.9)	0.6235
Coronary artery disease	12 (48)	23 (46.9)	0.9311
Prior sternotomy	14 (56)	32 (65.3)	0.4580
History of smoking	10 (40)	25 (51)	0.4625
*Donor characteristics*			
Age, years, median [IQR]	28 [25, 33]	33 [26, 39]	0.1026
Male, sex, *n* (%)	21 (84)	40 (81.6)	0.8002
Donor-recipient sex mismatch	7 (28)	11 (22.4)	0.5986
Height, m	1.762 (0.113)	1.764 (0.083)	0.9171
Weight, kg	85.1 (15.1)	91.9 (18.2)	0.1074
BMI, kg/m^2^	27.5 (5.2)	29.6 (6.2)	0.1522
Left ventricular ejection fraction	63.5 (6.4)	61.7 (6.4)	0.2410
Cause of death/mechanism of injury			0.5536
Anoxia	12 (48)	26 (53)	
Cerebrovascular accident	1 (4)	6 (12.2)	
Head trauma	11 (44)	16 (32.7)	
Other	1 (4)	1 (2.1)	

Abbreviations: DCD, donation after circulatory death; HCT, hematocrit; PGD, primary graft dysfunction.

**Table 2:** Procedure and Hemodynamic Characteristics

Variables	HCT < 15 (*n* = 25)	HCT ≥ 15 (*n* = 49)	*p*-value
*Procedure characteristics*			
Warm ischemic time, minutes	22 [19, 24]	21 [18, 25]	0.7658
OCS perfusion time, minutes	246 [232, 278]	254 [220, 290]	0.6353
Cardiopulmonary bypass time	199 [165, 214]	194 [167, 236]	0.9635
*Hemodynamic characteristics (24 hours post ICU admission)*			
Mean arterial pressure	74 [71, 84]	74.5 [70, 81.5]	0.9768
Heart rate	109 [108, 109]	109 [108, 110]	0.8740
Central venous pressure	11 [8, 13]	11.5 [9.5, 14]	0.2850
Mean pulmonary artery pressure	19 [15, 24]	20 [18, 23]	0.4521

Abbreviations: HCT, hematocrit; OCS, Organ Care System.

There were no significant differences in moderate or severe PDG incidence (*p =* 0.8654, odds ratio (OR) 0.90, 95% confidence interval (CI) 0.26-3.04), and ECMO utilization (HCT < 15%: 4 (16%) vs HCT ≥ 15%: 9 (18.37%); *p* = 0.8002) (Table 3). The odds of PGD (*p* = 0.8654, OR 0.90 95% CI 0.266-3.042) and ECMO requirement (*p* = 0.8003, OR 1.181 95% CI 0.325-4.295) were not significantly different between groups.

**Table 3:** Outcomes of Patients Following DCD Heart Transplantation Categorized by HCT Level

Outcomes	HCT < 15 (*n* = 25)	HCT ≥ 15 (*n* = 49)	*p*-value
PGD			0.8654
None	20 (80)	40 (81.6)	
Moderate	1 (4)	2 (4.1)	
Severe	4 (16)	7 (14.3)	
ECMO	4 (16)	9 (18.37)	0.8002
Post-transplant hospital length of stay, days	14 [11, 20]	19 [14, 26]	0.3719
Post-transplant intensive care unit length of stay, days	6 [5, 7]	8 [6, 12]	0.1042
Venous delta lactate	1.75 (1.14)	2.09 (1.41)	0.3534
Arterial delta lactate	1.80 (1.47)	1.91 (1.33)	0.7503
Inotrope score			
Admission	12.77 (5.47)	15.16 (7.77)	0.1734
48 hours after admission	5.79 (4.57)	10.64 (10.89)	*0.0368*
Postoperative complications	7 (35)	17 (39.5)	0.7301
Postoperative stroke	0 (0)	2 (4.6)	0.3270
Postoperative sepsis	1 (5)	4 (9.3)	0.5565
Postop renal failure	1 (7.7)	9 (34.6)	0.0695
Post-Op-ReOp bleeding	3 (15)	4 (9.3)	0.5030
Prolonged ventilation	10 (50)	20 (46.5)	0.7964
Rate of survival	25 (100)	44 (91.7)	0.9567

Abbreviations: DCD, donation after circulatory death; ECMO, extracorporeal machine oxygenation; HCT, hematocrit; PGD, primary graft dysfunction.

Italic p-value represents a statistically significant p-value based on an alpha of 0.05.

The mean inotrope score 48 hours post admission was significantly higher in DCD recipients with higher perfusate HCT (5.79 vs 10.64; *p* = 0.0368), although the inotrope score at admission was similar between the groups (Table 3). There was also a trend toward longer hospital (14 vs 19; *p* = 0.3719) and intensive care unit (6 vs 8; *p* = 0.1042) length of stay for the higher HCT group, though this difference did not reach statistical significance (Table 3). A similar trend was observed for postoperative renal dysfunction in the higher HCT cohort, although it did not reach statistical significance (Table 3).

## Discussion

**Figure 1:**
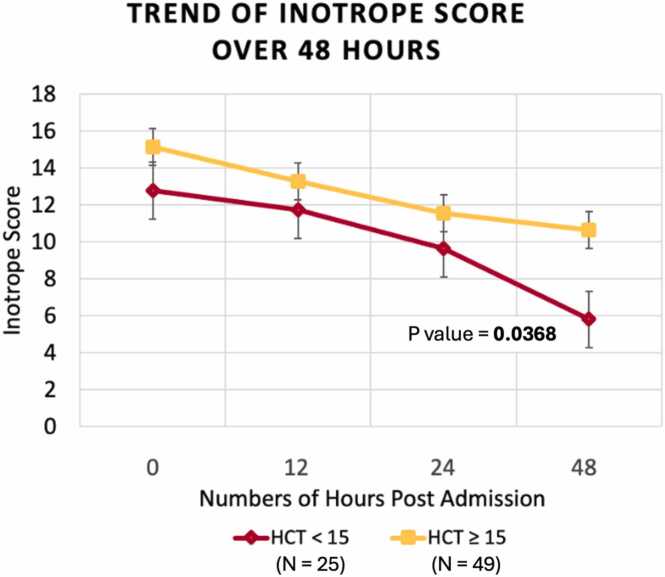
Graph showing the association between higher hematocrit levels during ex vivo heart perfusion and duration of inotrope use. HCT, hematocrit.

There is considerable variability in the HCT percentage among hearts perfused via direct procurement and perfusion using the Transmedics OCS. This analysis demonstrates that higher perfusate HCT during ex vivo machine is not associated with significant reductions in PGD or ECMO utilization but may correlate with elevated inotrope requirements post transplant (Figure 1). These findings raise valuable questions on the potential impact of perfusate composition on graft quality and patient outcomes.

These findings suggest that lower HCT levels during ex vivo heart perfusion—relative to physiologic levels in the recipient—might be protective for the transplanted heart. While the presence of RBCs in the perfusate contributes to oxygen delivery, the ideal HCT range remains undefined. Although vascular resistance is primarily influenced by vessel size, increased viscosity within the perfusate at higher HCT levels could contribute to reduced perfusion efficiency during ex vivo perfusion.^6^ Consequently, our higher HCT group may have increased viscosity, potentially leading to perfusion issues that become apparent only after transplantation.^7^ The observed trend toward higher postoperative renal failure in the higher HCT cohort may be part of the same physiologic process. Increased perfusate viscosity may impair microcirculatory flow, leading to subclinical myocardial ischemia and reduced early graft recovery. This may, in turn, compromise systemic perfusion and renal blood flow post-transplant, contributing to transient renal dysfunction. Higher HCT levels in the perfusate could also result in increased hemolysis-induced graft injury due to the introduction of hemolytic toxins.^8^, ^9^ These results contrast with findings from lung transplantation studies, where lower hemoglobin perfusate levels have been linked to a higher incidence of early allograft dysfunction, as well as juvenile porcine models of DCD heart perfusion, which showed suboptimal heart recovery and impaired function under low-hemoglobin conditions.^10^, ^11^ Furthermore, these findings could influence transplant protocols by potentially reducing the duration of blood collection, which adds to the WIT and the risk of introducing external toxins. Previous studies have demonstrated the benefits of minimizing WIT on graft health and survival.^12^, ^13^

Emerging evidence from other organ perfusion studies, such as in lungs and kidneys, suggests that acellular perfusates may reduce proinflammatory signaling while maintaining organ viability.^14^, ^15^, ^16^ Although cellular perfusates may afford increased length of preservation, their role in increasing graft injury warrants further investigation.^17^, ^18^, ^19^ Thus, an important factor to consider is the anticipated length of time the organ will be perfused when selecting a perfusate composition.

Limitations of this study include its retrospective single-center, which limits generalizability and the ability to establish strong causality. The relatively small sample size may have reduced statistical power. Moreover, the study may not have accounted for all potential confounding variables, such as variations in surgical techniques and postoperative management. For instance, variability in additional procedural data such as coronary flow rates, rate of adenosine infusion may influence outcomes and should be evaluated in further studies. Additionally, there is the technological limitation of classifying HCT values to either ≥15% PCV or <15% PCV based on i-STAT analysis reports because of nonspecific measurements for HCT values <15% PCV. Furthermore, echocardiographic and detailed hemodynamic parameters at 48 hours were not consistently collected in this cohort. The absence of these data limits physiologic interpretation and represents an important area for future prospective studies. Thus, while these results provide preliminary insights into the impact of perfusate HCT on postoperative transplant outcomes, this study is primarily hypothesis-generating and would necessitate larger prospective studies needed to establish causality and inform clinical guidelines.

## Conclusion

Higher perfusate HCT level during ex vivo perfusion of DCD hearts does not appear to protect against PGD or ECMO utilization post transplant but may be associated with higher inotrope requirements and longer ICU stays. These findings raise important questions about optimal HCT levels for ex vivo perfusion and further exploration of perfusate composition for DCD heart transplantation.

## Declaration of Competing Interest

The authors declare that they have no known competing financial interests or personal relationships that could have appeared to influence the work reported in this paper.
